# Evaluating the Reliability of Expert Evidence in Compensation Procedures: Are Diagnosticians Influenced by the Narrative Fallacy when Assessing the Psychological Injuries of Trauma Victims?

**DOI:** 10.1007/s12207-016-9263-5

**Published:** 2016-07-14

**Authors:** M. J. J. Kunst, M. Van de Wiel

**Affiliations:** Faculty of Law, Institute for Criminal Law and Criminology, Leiden University, Room C1.03, P.O. Box 9520, 2300 RA Leiden, The Netherlands

**Keywords:** Compensation, Psychological injury, Trauma, Psychodiagnostic assessment, Narrative fallacy

## Abstract

The current study investigated whether mental health practitioners are influenced by the narrative fallacy when assessing the psychological injuries of trauma victims. The narrative fallacy is associated with our tendency to establish logical links between different facts. In psychodiagnostic assessments, this tendency may result in overdiagnosis of mental disorders when psychological symptoms can be attributed to a traumatic event. Consequently, legal decision makers may be at risk of awarding compensation for psychological injuries which are not severe enough to justify financial reimbursement. To explore this topic, we asked Dutch mental health practitioners whether they would assign a diagnosis of mental disorder to fictitious symptoms of psychological injury. Each participant was presented with two vignettes. The first vignette described symptoms in terms of a generalized anxiety disorder; the second in terms of a major depressive episode. The vignettes varied in the cause (trauma versus cause not specified) and severity (near threshold of DSM diagnosis versus below threshold of DSM diagnosis) of the symptoms. Results indicated that participants more often assigned a diagnosis of mental disorder if the psychological symptoms had been caused by a traumatic event than if that had not been the case. Further analysis of the data suggested that this difference was due to the high numbers of assigned diagnoses of posttraumatic stress and acute stress disorder in the trauma conditions. It was speculated that participants filled in missing information to justify the assignment of such diagnoses, for example by imagining symptoms of intrusion and avoidance.

## Introduction

The psychological impact of trauma may play an important role in compensation procedures. Partly encouraged by international and supranational obligations, the majority of Western countries have ensured that trauma victims can claim compensation for psychological injury through a number of different routes. In the Netherlands, for example, trauma victims can claim compensation for psychological injury through the criminal justice system (in case of a criminal incident), a civil lawsuit, or a disability compensation scheme. Although the legal criteria to award a request for compensation differ between these compensation modalities, each modality allows victims to claim financial compensation for material damages, such as costs for the treatment of psychological problems, temporary sick leave, or permanent work disability, and non-material damages, such as the pain and suffering characteristic of psychological injury. However, trauma victims are usually only eligible for compensation if their psychological problems are recognized as a mental disorder by the Diagnostic and Statistical Manual of Mental Disorders (DSM; American Psychiatric Association, 2013) or another widely accepted diagnostic classification system (see Kunst, 2014).

### Evaluating a Claim for Psychological Injury Compensation

When evaluating a claim for financial compensation for psychological injury, legal decision makers often rely on information provided by a psychological expert (Cutler & Kovera, 2011). Although several authors have argued that therapists should refrain from engagement in forensic evaluations (e.g., Strasburger, Gutheil, & Brodsky, 1997), this is usually a psychologist or psychiatrist who treats the victim for his or her psychological injuries. Only when information from a therapist is lacking or doubted, an independent psychological expert is consulted. However, unfortunately, several sources of bias may question the trustworthiness of psychological practitioners’ diagnostic information. In particular, the use of *heuristics* during the diagnostic decision process may result in erroneous conclusions about a victim’s psychological injury. Heuristics are “strategies that ignore part of the information (or the lack of information), with the goal of making decisions more quickly, frugally, and/or accurately than more complex methods” (Gigerenzer & Gaissmaier, 2011, p. 454). In this study, we will focus on one specific heuristic: the narrative fallacy. Taleb (2007, pp. 63–64) provides the following description of the narrative fallacy:

The narrative fallacy addresses our limited ability to look at sequences of facts without weaving an explanation into them, or, equivalently, forcing a logical link, an arrow of relationship upon them. Explanations bind facts together. They make them all the more easily remembered; they help them make more sense. Where this propensity can go wrong is when it increases our impression of understanding.

This definition suggests that we always want to explain why things happen. This characteristic helps us to create a plausible story. However, there is a second characteristic of the narrative fallacy which is not included in this definition: We sometimes make up things to fill in missing information or neglect information that does not fit in with our story (for an example, see Thüring, Großmann, & Wender, 1985). Consequently, we sometimes base our conclusions on a wrong story (Menashe & Shamash, 2005).

### The Narrative Fallacy in Psychodiagnostic Assessments

The narrative fallacy may also play a role in psychodiagnostic assessments: If psychological symptoms can be linked to a particular event, psychologists or psychiatrists may be more likely to attribute these symptoms to that event. Empirical studies which addressed this topic are, however, rather scarce. A few exceptions can be found in the work of Kim and colleagues. For example, in an experimental vignette study, Kim and Ahn (2002) asked clinical psychologists to formulate causal theories about fictitious patients’ psychological symptoms and found that a traumatic event was thought to be an important cause of panic attacks in a phobic situation and excessive fear in response to a specific object or situation. In another experimental vignette study, Kim and colleagues investigated how causal information affects clinical psychologists’ tendency to assign a diagnosis of major depressive disorder (MDD; Kim, Paulus, Nguyen, & Gonzalez, 2012). More specifically, they investigated whether clinical psychologists withhold clients a diagnosis of MDD if their symptoms begin within 2 months of the loss of a loved one and do not persist beyond these 2 months. The fourth revised version of the Diagnostic and Statistical Manual of Mental Disorders (DSM-IV-TR, American Psychiatric Association, 2000) advised, though not obliged, clinicians to refrain from a diagnosis of MDD in case of a bereavement-related life event. Results suggested that clinical psychologists were indeed less likely to assign a diagnosis of MDD when symptoms followed upon the experience of a stressful life event than when no or a neutral event had occurred: When a stressful life event had occurred, a diagnosis of MDD was less advocated than when such an event had not occurred. However, this applied to both bereavement-related and non-bereavement-related life events. This finding may be explained by what Meehl (1973) has called *the understanding-it-makes-it-normal effect*. This effect refers to people’s tendency to rate other people’s behaviors as less abnormal when they know the cause of those behaviors. In line with this effect, Kim, Paulus, Nguyen, et al. (2012) also found that abnormality ratings were lower when depression symptoms could be ascribed to a stressful life event.^1^ Finally, again in an experimental vignette study, Kim, Paulus, Gonzalez, and Khalife (2012) found that clinical psychologists’ tendency to rate symptoms of MDD or posttraumatic stress disorder (PTSD) as *abnormal* depended on the proportionality of a subject’s psychological response to a particular stressful event; Severe symptom levels were considered less abnormal in response to a traumatic event than in response to a mildly distressing event.

Based on the work of Kim and colleagues, it seems reasonable to speculate that the narrative fallacy may indeed play a role in psychodiagnostic decision-making. However, their findings do not indicate *how* this fallacy affects clinicians’ tendency to assign a diagnosis of mental disorder based on a formal classification system, such as DSM. On the one hand, their results suggest that clinicians are less likely to diagnose people with a formal mental disorder if symptoms are caused by a particular event. This fits in with the first characteristic of the narrative fallacy: our tendency to explain why things happen. On the other hand, the designs of their studies do not allow such a conclusion to be drawn. First, their studies included relatively low numbers of clinical psychologists (*N*s < 80). It can therefore not be ruled out that their results were biased by sample size. Second, and more important, they either requested participants to indicate whether they advocated a particular diagnosis, such as MDD, or to rate symptoms in terms of abnormality. It can therefore not be ruled out that participants would have responded differently to a question which allowed them to advocate *any* diagnosis of a mental disorder. Particularly, it cannot be ruled out that participants would have assigned a diagnosis of a trauma- or stressor-related disorder (i.e., a diagnosis of acute stress disorder [ASD], adjustment disorder [AD], PTSD, or reactive attachment disorder) more often in the trauma than in the non-trauma conditions. For this type of disorders, exposure to a stressful event is a diagnostic requirement. Consequently, such an event cannot serve as an indication of the normality of experienced symptoms. Rather, it may serve as a justification to assign a diagnosis of a trauma- or stressor-related disorder. Indeed, it might even be the case that this feature triggers the tendency to fill in gaps of missing information or to neglect information—the second characteristic of the narrative fallacy. For example, if psychological symptoms are presented in combination with a traumatic event, psychodiagnostic decision makers may fill in missing symptoms or neglect unfitting information to assign a diagnosis of a trauma- or stressor-related disorder. However, these contentions have never been tested empirically.

### The Current Study

Given the aforementioned, the current study examined the narrative fallacy in psychodiagnostic assessments conducted by Dutch mental health practitioners. More specifically, we investigated whether the outcomes of psychodiagnostic assessments are affected by cues about the potential cause of the psychological injury. This is particularly important from a compensation perspective. On the one hand, if diagnoses of mental disorder are more frequently assigned to psychological injuries that can be related to a particular event, then legal decision makers may be at risk of awarding compensation for psychological injuries which do not qualify for a diagnosis of mental disorder according to DSM or another diagnostic classification system. On the other hand, if diagnoses of mental disorder are less frequently assigned to psychological injuries that can be related to a particular event, then legal decision makers may be at risk of withholding compensation for psychological injuries which do qualify for a diagnosis of mental disorder according to DSM or another diagnostic classification system. Both outcomes are unwelcome and should therefore be taken into account in compensation procedures.

## Methods

### Participants

Participants were mental health care practitioners who were registered in Vektis. This is a digital database and includes, among other things, contact details of most health care providers in the Netherlands. This database can be consulted via www.vektis.nl. At the time of our data collection, 11,964 mental health care practitioners were registered in this database with an e-mail address (≈75 % of all registrants). Of these, 2369 (19.8 %) agreed to participate in our research. Each of them was randomly assigned to either of two substudies: the current study or a study about another fallacy (i.e., other than the narrative fallacy) that can bias the outcome of a diagnostic assessment. Of those assigned to the current study (*n* = 1282), 1154 (90.0 %) provided complete responses on questions about background characteristics and responded to at least one vignette. The large majority of respondents were female (*n* = 907, 78.6 %), had the Dutch nationality (*n* = 1096, 95.0 %), worked as a mental health psychologist (*n* = 807, 69.9 %) and were employed by a mental health institution (*n* = 637, 55.2 %). On average, they were 46.6 years old (*SD* = 11.9).

### Procedure and Materials

Each participant was presented two vignettes: one about a 35-year-old man who suffered from symptoms of generalized anxiety disorder (GAD; vignette 1) and one about a 55-year-old woman who suffered from symptoms of a major depressive episode (MDE; vignette 2). Vignette descriptions were obtained from two previous studies about the impact of payment method on psychologists’ diagnostic decisions (Kielbasa, Pomerantz, Krohn, & Sullivan, 2004; Pomerantz & Segrist, 2006,2) and were translated in Dutch. Participants were randomly assigned to one of four conditions: (1) symptom level near threshold of DSM diagnosis (severe symptom level), a victim of a traumatic event (trauma); (2) symptom level near threshold of DSM diagnosis (severe symptom level), not a victim of a traumatic event (non-trauma); (3) symptom level below threshold of DSM diagnosis (moderate symptom level), a victim of a traumatic event (trauma); or (4) symptom level below threshold of DSM diagnosis (moderate symptom level), not a victim of a traumatic event (non-trauma). For the two trauma conditions, the vignette descriptions were slightly extended to indicate that symptoms had started after a violent street robbery. For each vignette, participants had to indicate whether they would assign a diagnosis according to DSM (“yes” or “no”) if the person described in the vignette would come to them for therapy. Those who answered “yes” to this question were additionally asked to indicate what specific diagnosis they would assign. This question could be answered by filling in a blank space. To ensure that the order of vignettes would not affect our results, we randomly varied the order of vignettes in each condition.

Support for the first characteristic of the narrative fallacy would be delivered if participants in the trauma conditions more frequently assigned a DSM diagnosis than participants in the non-trauma conditions. If an eventual difference would be due to a large number of diagnoses of ASD and PTSD, then support for the second characteristic of the narrative fallacy would also be delivered. Symptoms of GAD or MDE may fulfill the criteria for a diagnosis of adjustment disorder, but not for a diagnosis of ASD or PTSD. After all that would require the presence of additional symptoms, particularly symptoms of intrusion and avoidance. Assigning a diagnosis of ASD or PTSD would therefore indicate that participants imagined additional symptoms.

### Statistical Analyses

For both vignettes, we performed a series of three chi-square tests. We first compared the proportions of assigned DSM diagnoses between respondents in the trauma conditions and those in the non-trauma conditions, irrespective of symptom level. Then, we compared the proportions of assigned DSM diagnoses between respondents in the two severe symptom level conditions (trauma versus non-trauma) and between respondents in the two moderate symptom level conditions (trauma versus non-trauma). To reduce the likelihood of making type I errors, we set the alpha level at *p* < 0.0083 to account for the number of comparisons (*p* < 0.0083 [*p* < 0.05/6]). Finally, we calculated the frequencies and corresponding percentages of assigned DSM diagnoses. This would give us an idea of differences in types of assigned diagnoses between the different vignettes and conditions.

## Results

### Differences in Proportions of Assigned DSM Disorders Between the Four Experimental Conditions

As can be seen from Tables 1 and 2, respondents in the trauma conditions assigned DSM diagnoses more often than those in the non-trauma conditions. For both vignettes, this difference was significant: *χ*^2^ (1, *N* = 1096) = 47.58, OR = 2.36, 95 % CI = 1.85–3.02, *p* < 0.001 for vignette 1 (generalized anxiety disorder) and *χ*^2^ (1, *N* = 1079) = 40.53, OR = 2.21, 95 % CI = 1.73–2.82, *p* < 0.001 for vignette 2 (major depressive episode*)*. Moreover, it did not seem to matter whether respondents had read a vignette about severe or moderate symptom levels. In both cases, participants in the trauma conditions had assigned a DSM diagnosis more often than those in the non-trauma conditions: *χ*^2^ (1, *N* = 546) = 4.21, OR = 1.49, 95 % CI = 1.02–2.18, *p* = 0.04 for vignette 1/severe symptom level; *χ*^2^ (1, *N* = 525) = 16.60, OR = 2.13, 95 % CI = 1.48–3.08, *p* < 0.001 for vignette 2/severe symptom level; *χ*^2^ (1, *N* = 550) = 57.98, OR = 3.89, 95 % CI = 2.73–5.56, *p* < 0.001 for vignette 1/moderate symptom level; and *χ*^2^ (1, *N* = 554) = 32.78, OR = 3.29, 95 % CI = 2.16–5.00, *p* < 0.001 for vignette 2/moderate symptom level. However, given the more stringent alpha level of *p* < 0.0083, for vignette 1 this difference was not significant for respondents in the severe symptom level condition. Overall, these results provide substantial support for the first characteristic of the narrative fallacy; the tendency to establish a logical link between different facts.

**Table 1:** Trauma victimization by DSM diagnosis—vignette 1 (generalized anxiety disorder)

		DSM diagnosis												
		Yes						No						
		Moderate		Severe		Total		Moderate		Severe		Total		
		*n*	%	*n*	%	*n*	%	*n*	%	*n*	%	*n*	%	Total
Trauma	Yes	164	67.8	215	53.8	379	59.0	108	35.1	64	43.8	172	37.9	551
	No	78	32.2	185	46.3	263	41.0	200	64.9	82	56.2	282	62.1	545
	Total	242	100	400	100	642	100	308	100	146	100	454	100	1096

Moderate = moderate symptom level. Severe = severe symptom level

**Table 2:** Trauma victimization by DSM diagnosis—vignette 2 (major depressive episode)

		DSM diagnosis												
		Yes						No						
		Moderate		Severe		Total		Moderate		Severe		Total		
		*n*	%	*n*	%	*n*	%	*n*	%	*n*	%	*n*	%	Total
Trauma	Yes	97	71.3	196	57.1	293	61.7	180	43.1	70	38.5	250	42.2	543
	No	39	28.7	147	42.9	186	38.3	238	56.9	112	61.5	350	57.8	536
	Total	136	100	343	100	479	100	418	100	182	100	600	100	1079

Moderate = moderate symptom level. Severe = severe symptom level

### Differences in Types of Assigned DSM Disorders Between the Four Experimental Conditions

A closer look at respondents’ diagnostic decisions revealed that approximately 30 % of them were not willing to assign a definite diagnosis based on the information provided by the vignettes. This applied to both vignettes and to severe as well as moderate symptom levels (see Tables 3 and 4). In addition, some differences appeared to exist between the trauma and non-trauma conditions in respondents’ tendency to assign a particular type of diagnosis. In the trauma conditions, the number of assigned diagnoses of ASD and PTSD was much higher than that in the non-trauma conditions (188 versus 1 for vignette 1 and 136 versus 0 for vignette 2). Conversely, the number of assigned diagnoses of anxiety and mood disorders was much higher in the non-trauma than that in the trauma conditions (147 versus 37 for vignette 1 and 112 versus 35 for vignette 2). However, the differences in numbers of assigned diagnoses of anxiety and mood disorders between the non-trauma and trauma conditions were not as large as the differences in the numbers of assigned diagnoses of ASD and PTSD between the trauma and non-trauma conditions (110 versus 187 for vignette 1 and 77 versus 136 for vignette 2). Although we need to be cautious in not making the narrative fallacy ourselves, this might explain why respondents in the trauma conditions were more likely to assign a diagnosis of mental disorder than those in the non-trauma conditions. Indeed, if, in the non-trauma conditions, 77 (187 − 110) more respondents had assigned a diagnosis of mental disorder to the symptoms described in vignette 1 and if 59 (136 − 77) more respondents had assigned a diagnosis of mental disorder to the symptoms described in vignette 2, then the differences in proportions of assigned diagnoses between the trauma and non-trauma conditions would have become insignificant. Finally, it might be speculated that this result also provides some support for the second characteristic of the narrative fallacy; the tendency to fill in gaps of missing information or to neglect information to enable the establishment of a logical link between different facts. After all, the two vignettes did not provide enough information to justify a diagnosis of ASD or PTSD.

**Table 3:** Trauma victimization by diagnostic category—vignette 1 (generalized anxiety disorder)

			Diagnostic category																
			ASD		PTSD		AD		Anxiety		Mood		Other		INFO		NS		
			*n*	%	*n*	%	*n*	%	*n*	%	*n*	%	*n*	%	*n*	%	*n*	%	Total
Moderate	Trauma	Yes	2	1.2	80	48.8	18	11.0	12	7.3	0	0.0	3	1.8	43	26.2	6	3.7	164
		No	0	0.0	0	0.0	6	7.7	39	50.0	3	3.8	5	6.4	21	26.9	4	5.1	78
		Total	2	0.8	80	33.1	24	9.9	51	21.1	3	1.2	8	3.3	64	26.4	10	4.1	242
Severe	Trauma	Yes	1	0.5	105	48.8	3	1.4	25	11.6	0	0.0	2	0.9	68	31.6	11	5.1	215
		No	1	0.5	0	0.0	4	2.2	105	56.8	0	0.0	6	3.2	57	30.8	12	6.5	185
		Total	2	0.5	105	26.3	7	1.8	130	32.5	0	0.0	8	2.0	125	31.3	23	5.8	400

Moderate = moderate symptom level. Severe = severe symptom level

*ASD* acute stress disorder, *PTSD* posttraumatic stress disorder, *AD* adjustment disorder, *Anxiety* anxiety disorder, *Mood* mood disorder, *Other* other diagnosis, *INFO* not enough information to specify diagnosis, *NS* diagnosis not specified

**Table 4:** Trauma victimization by diagnostic category—vignette 2 (major depressive episode)

			Diagnostic category																
			ASD		PTSD		AD		Anxiety		Mood		Other		INFO		NS		
			*n*	%	*n*	%	*n*	%	*n*	%	*n*	%	*n*	%	*n*	%	*n*	%	Total
Moderate	Trauma	Yes	9	9.3	38	39.2	14	14.4	2	2.1	11	11.3	2	2.1	16	16.5	5	5.2	97
		No	0	0.0	0	0.0	1	2.6	0	0.0	25	64.1	1	2.6	6	15.4	6	15.4	39
		Total	9	6.6	38	27.9	15	11.0	2	1.5	36	26.5	3	2.2	22	16.2	11	8.1	136
Severe	Trauma	Yes	1	0.5	88	44.9	3	1.5	3	1.5	19	9.7	1	0.5	76	38.8	5	2.6	196
		No	0	0.0	0	0.0	0	0.0	0	0.0	87	59.2	11	7.5	43	29.3	6	4.1	147
		Total	1	0.3	88	25.7	3	0.9	3	0.9	106	30.9	12	3.5	119	34.7	11	3.2	343

Moderate = moderate symptom level. Severe = severe symptom level

*ASD* acute stress disorder, *PTSD* posttraumatic stress disorder, *AD* adjustment disorder, *Anxiety* anxiety disorder. *Mood* mood disorder, *Other* other diagnosis, *INFO* not enough information to specify diagnosis, *NS* diagnosis not specified

## Discussion

Diagnostic assessments of psychological injury may play an important role in legal procedures about compensation for psychological injury following trauma. Unfortunately, the current study suggests that such assessments may be biased by the narrative fallacy: Just as anyone else, mental health care practitioners appear to make stories to explain why certain things happen by establishing logical links between certain facts. Those who participated in our study were more likely to assign a diagnosis of mental disorder if they had read a vignette which contained information about the possible cause of the psychological symptoms than if they had read a vignette which did not contain such information. Moreover, this finding did not depend on the nature or severity of presented symptoms. In other words, assigning a diagnosis of mental disorder is more logical when symptoms can be linked to a particular cause. Inspection of the types of diagnoses suggested that the relative difference in assigned diagnoses between the trauma and non-trauma conditions may have been due to diagnosticians’ tendency to fill in missing information to create a symptom picture that justifies a diagnosis of ASD or PTSD.

Our results suggest that legal decision makers should be cautious in relying on psychological experts’ opinions about their clients’ mental health status. Since psychological experts are more likely to assign a diagnosis of mental disorder if symptoms can be attributed to a particular cause, some trauma victims may fulfill the criteria for compensation, although their symptoms are actually not severe enough to justify compensation for psychological injury.

To avoid erroneous decisions about compensation, legal decision makers should confront the expert with the possibility of the narrative fallacy by asking him or her how he or she would classify the victim’s symptoms if these had not been triggered by a traumatic event. This might particularly be worthwhile if the expert’s report does not explicitly mention that the victim suffers from symptoms which are typical for a trauma- or stressor-related disorder, but a diagnosis of such a disorder is nevertheless assigned. In cases where the stakes are high (e.g., when high amounts of compensation are requested), it might even be worthwhile to have two or more independent experts comment upon the initial expert’s opinion without letting them know that the symptoms to be evaluated have been caused by a traumatic event and are the subject of a compensation claim. This strategy is rather laborious and may not always be feasible, because it requires that references to the traumatic event are deleted from the initial expert’s report. However, if this is possible and if the independent experts come to another conclusion about the classification of the victim’s symptoms, then this may be an indication that the initial expert’s diagnostic conclusions suffered from the narrative fallacy. After all, without information about the potential cause of the psychological symptoms, independent experts are probably less prone to making the narrative fallacy themselves.

Despite the aforementioned, caution should be taken when interpreting our results and speculating about their practical implications. After all, our study had several limitations. First, we did not investigate whether wrongly assigned diagnoses of mental disorders indeed result in bad decisions about compensation. Our respondents were told that the person described in the vignette came to them for therapy. It cannot be ruled out that legal decision makers already more critically evaluate information from psychological experts who also treat the litigating victim than information from independent experts. The former may be seen as *hired guns*—experts who simply take the position of their client in return for payment (Ziemke & Brodsky, 2015). Several studies suggest that the opinion of such experts is often considered with suspicion by legal decision makers (see Edens, Smith, Magyar, Mullen, Pitta, & Petrila, 2012; Mossman, 1999). Second, we obtained a rather low response rate. This questions the generalizability of our results to the entire population of mental health practitioners in the Netherlands. Third, vignettes differ from real-life diagnostic settings. Participants who assigned a diagnosis to the persons described in our vignettes would perhaps have made another decision if they had been able to perform a full-blown diagnostic assessment.

To conclude, our study was the first to investigate the narrative fallacy in a large sample of mental health practitioners. Future studies might build upon our study by overcoming its limitations and by addressing several additional issues. In particular, it would be interesting to see whether our results extend to other types of trauma than violent crimes, because type of trauma may strengthen or weaken diagnosticians’ tendency to assign a diagnosis of mental disorder if symptoms can be explained by a traumatic event. Based on previous research, it may be suggested that it makes a difference whether someone is hurt intentionally (as in our study) or accidentally. For example, in a series of experiments, Ames and Fiske (2013) found that intentional harms are considered as more severe than unintentional harms, also in terms of monetary values, even if events do not differ in actual harm. Building upon this study, it would be worthwhile to see whether diagnosticians’ decisions to assign a diagnosis of mental disorder vary by the intentionality of suffered harm.

## Footnotes

1 Similar findings were found among undergraduate students who were enrolled in an introductory psychology course (Ahn, Novick, & Kim, 2003; Kim & LoSavio, 2009).

2 These references provide the unadapted full texts of the vignettes we used.

## References

[CR1] Ahn W-K, Novick L, Kim N (2003). Understanding behavior makes it more normal. Psychonomic Bulletin & Review.

[CR2] American Psychiatric Association. (2000). *Diagnostic and statistical manual of mental disorders* (4th ed., text rev.). Washington, DC: Author.

[CR3] American Psychiatric Association (2013). Diagnostic and statistical manual of mental disorders.

[CR4] Ames DL, Fiske ST (2013). Intentional harms are worse, even when they’re not. Psychological Science.

[CR5] Association of Universities in the Netherlands (2014). *The Netherlands Code of Conduct for Scientific Practice: Principles of good scientific teaching and research. Author: Amsterdam*. Retrieved May 13, 2016, from http://www.vsnu.nl/files/documenten/Domeinen/Onderzoek/The_Netherlands_Code%20of_Conduct_for_Academic_Practice_2004_(version2014).pdf

[CR6] Cutler BL, Kovera MB (2011). Expert psychological testimony. Current Directions in Psychological Science.

[CR7] Edens J, Smith S, Magyar M, Mullen K, Pitta A, Petrila J (2012). “Hired guns”, “charlatans”, and their “voodoo psychobabble”: case law references to various forms of perceived bias among mental health expert witnesses. Psychological Services.

[CR8] Gigerenzer G, Gaissmaier W (2011). Heuristic decision making. Annual Review of Psychology.

[CR9] Kielbasa A, Pomerantz A, Krohn E, Sullivan B (2004). How does clients’ method of payment influence psychologists’ diagnostic decisions?. Ethics & Behavior.

[CR10] Kim N, Ahn W-K (2002). Clinical psychologists’ theory-based representations of mental disorders predict their diagnostic reasoning and memory. Journal of Experimental Psychology: General.

[CR11] Kim NS, LoSavio ST (2009). Causal explanations affect judgments of the need for psychological treatment. Judgment and Decision Making.

[CR12] Kim NS, Paulus DJ, Gonzalez JS, Khalife D (2012). Proportionate responses to life events influence clinicians’ judgments of psychological abnormality. Psychological Assessment.

[CR13] Kim NS, Paulus DJ, Nguyen TP, Gonzalez JS (2012). Do clinical psychologists extend the bereavement exclusion for major depression to other stressful life events?. Medical Decision Making.

[CR14] Kunst MJJ (2014). Simulatie onder slachtoffers van schokkende gebeurtenissen: Een pleidooi voor onafhankelijk onderzoek naar de echtheid van psychische klachten in schadevergoedingsprocedures (Faking among victims of high-impact incidents: A plea for independent assessment of malingering compensation procedures). Recht der Werkelijkheid.

[CR15] Meehl PE, Meehl PE (1973). Why I do not attend case conferences. Psychodiagnosis: Selected papers.

[CR16] Menashe, D., & Shamash, M. (2005). The narrative fallacy. *International Commentary on Evidence, 3*(1). doi:10.2202/1554-4567.1034

[CR17] Mossman D (1999). “Hired guns,” “whores,” and “prostitutes”: case law references to clinicians of ill repute. The Journal of the American Academy of Psychiatry and the Law.

[CR18] Pomerantz A, Segrist D (2006). The influence of payment method on psychologists’ diagnostic decisions regarding minimally impaired clients. Ethics & Behavior.

[CR19] Strasburger LH, Gutheil TG, Brodsky A (1997). On wearing two hats: role conflict in serving as both psychotherapist and expert witness. The American Journal of Psychiatry.

[CR20] Taleb NN (2007). The Black Swan: The impact of the highly improbable.

[CR21] Thüring M, Großmann I, Wender KF, Gert R, Hans S (1985). Causal and temporal inferences and their effects on memory for discourse. Advances in psychology: Vol. 29. Inferences in text processing.

[CR22] Ziemke MH, Brodsky SL (2015). Unloading the hired gun: inoculation effects in expert witness testimony. International Journal of Law and Psychiatry.

